# Minimal clinically important differences in six-minute walking distance in late-onset Pompe disease

**DOI:** 10.1186/s13023-024-03156-3

**Published:** 2024-04-11

**Authors:** Kristl G. Claeys, Hani Kushlaf, Syed Raza, Noemi Hummel, Simon Shohet, Ian Keyzor, Agnieszka Kopiec, Ryan Graham, Brian Fox, Benedikt Schoser

**Affiliations:** 1 Department of Neurology, University Hospitals Leuven, Leuven, Belgium; 2 Department of Neurosciences, Laboratory for Muscle Diseases and Neuropathies, KU Leuven, Leuven Brain Institute (LBI), Leuven, Belgium; 3 University of Cincinnati College of Medicine, Cincinnati, OH USA; 4 Amicus Therapeutics, Ltd., Marlow, UK; 5 Certara GmbH, Lörrach, Germany; 6 Certara, Krakow, Poland; 7 Amicus Therapeutics, Inc., Princeton, NJ USA; 8 Department of Neurology, Friedrich-Baur-Institute, Ludwig-Maximilians-University, Munich, Germany

**Keywords:** Minimal clinically important difference, MCID, Six-minute walk distance, 6MWD, Late-onset Pompe disease, Disease severity

## Abstract

**Background:**

The minimal clinically important difference (MCID) is the smallest change in outcome that physicians or patients would consider meaningful and is relevant when evaluating disease progression or the efficacy of interventions. Studies of patients with late-onset Pompe disease (LOPD) have used the 6-min walk distance (6MWD) as an endpoint to assess motor function. However, an MCID for 6MWD (% predicted and meters) has yet to be established in LOPD. The objective of the study was to derive 6MWD MCID (% predicted and meters) with different analysis methods and for subgroups of different disease severity for LOPD.

**Methods:**

Data from the PROPEL trial were used to calculate 6MWD MCID in the overall PROPEL population and subgroups of baseline severity as assessed by walking distance and body mass index (BMI), using anchor- and distribution-based approaches.

**Results:**

The 6MWD MCIDs varied widely, depending on the method and subgroup, ranging from 2.27%-8.11% predicted for the overall LOPD population (23.7 m-57.2 m). For patients with baseline 6MWD < 150 m, MCIDs ranged from -0.74%-3.37% (-2.1 m-11.3 m). MCIDs increased with distance walked at baseline until a plateau was reached. For BMI subgroups, the MCIDs were generally lowest in obese patients.

**Conclusion:**

Our analysis shows that MCID depends on the chosen method and disease severity. The findings suggest that applying a single MCID to all patients can be misleading; consequently, a range of possible MCIDs should be considered. This may also be highly relevant for other neuromuscular diseases. This study provides a range of 6MWD MCIDs for LOPD, with lower MCIDs for more severe patients.

**Supplementary Information:**

The online version contains supplementary material available at 10.1186/s13023-024-03156-3.

## Introduction

The minimal clinically important difference (MCID) establishes a threshold for outcomes (either physician-measured or patient-reported) over which a patient would consider a given change in score to be meaningful, which is relevant when evaluating the efficacy of interventions during approval and reimbursement. The MCID is the smallest difference in score in the domain of interest that patients perceive as beneficial or harmful and which would mandate, in the absence of troublesome side effects and excessive cost, a change in the patient’s management [1]. The present study focuses on the MCID for improvement, knowing that the MCID for deterioration is precisely the reverse.

There are 3 main approaches to determining the MCID of an outcome measure: anchor-based, distribution-based, and Delphi [2–5]. The anchor-based approach uses external indicators to establish an MCID for the outcome measure. These anchors typically assess the patient’s perceived change between 2 time points, with response options such as “much better,” “a little better,” “unchanged,” “a little worse,” and “much worse.” The anchor must be positively correlated with the outcome measure for which the MCID is to be derived. Different anchors can provide different MCIDs. There is also a risk of recall bias because this approach relies on patients’ or clinicians’ perspectives [4, 6]. The anchor-based approach requires patient-level data from one or several studies. The distribution-based approach, on the other hand, uses statistical characteristics, e.g., standard deviation (SD), standard error measurement, effect size, and standardised response mean and relies on summary-level data, typically from a single study [3]. The Delphi approach requires selecting a panel of experts. Since the MCID is subject to variation depending on the method, disease, and clinical outcome, this opinion-based approach can help determine MCID values by consensus using questionnaires or surveys. This approach’s limitation is the experts’ subjectivity [3, 6].

Different approaches can result in different MCIDs being estimated [5], and MCIDs can differ depending on the (sub-)population being considered [7] or may vary with baseline values of the outcome measure for which the MCID is determined [8]. The 6-min walk distance (6MWD) assesses patients’ endurance and functional ability, monitors treatment efficacy and disease progression, and has been used in a range of diseases [9, 10]. Evidence of the MCID for 6MWD in meters has been reported in systematic literature reviews for various diseases, including cardiovascular, muscular, neuromuscular, and respiratory diseases [10, 11]. For example, the MCID for 6MWD for Duchenne muscular dystrophy ranged from 26.4 to 31.7 m, depending on the method used [10]. Most studies have reported 6MWD in meters as the primary clinical outcome measure, but 6MWD (% predicted) provides an accurate reflection of the disease as it considers physiological factors of an individual such as gender, age, height, and weight [12].

Late-onset Pompe disease (LOPD) is a rare autosomal recessive neuromuscular disorder, also known as glycogen storage disease type II, that causes progressive skeletal muscle damage [13, 14]. Studies of patients with LOPD have applied 6MWD to assess patients’ motor function during disease progression or treatment. However, the MCID for this endpoint in LOPD has not yet been established [15], which poses a challenge for clinicians, regulators, and Health Technology Assessment (HTA) bodies. This paper aims to derive MCIDs for 6MWD (% predicted and meters) for LOPD severities applying anchor-based and distribution-based methods, using data from a phase 3 randomized controlled trial.

## Methods

### Patient selection

To derive anchor- and distribution-based MCIDs for 6MWD for patients with LOPD, data from the PROPEL study were used [16]. PROPEL (NCT03729362) was a global, randomized, double-blind, parallel-group phase 3 trial that evaluated the efficacy and safety of cipaglucosidase alfa plus miglustat (*n* = 85) compared to alglucosidase alfa plus placebo (*n* = 38) in adult patients (age ≥ 18 years, body weight ≥ 40 kg) with confirmed LOPD. Patients had either never been treated with enzyme-replacement therapy (ERT-naive) or had been treated with alglucosidase alfa for ≥ 2 years (20 mg/kg once every 2 weeks; ERT-experienced). PROPEL was approved by independent ethics committees and institutional review boards at each site and was conducted by international guidelines for clinical studies such as the Declaration of Helsinki and Good Clinical Practice Guidelines. Additional study protocol details have been published previously [16].

### Data collection

In this study, we included the following outcomes that were measured in PROPEL:

(i) 6-min walk distance (6MWD): the distance (in meters) a patient can quickly walk within 6 min on a flat surface with walking shoes; walking aids (e.g., a cane, walker, or rollator) were permitted and were used consistently throughout the study, when required.
(ii) 6MWD % predicted: the actual distance walked in 6 min in meters divided by the predicted meters walked in 6 min of a healthy subject of comparable age, sex, height, and weight, as determined by the prediction equations from Enright and Sherrill, 1998 [12].
(iii) Forced vital capacity (FVC) % predicted [16, 17]: the volume of a maximal forced expiratory effort (FVC) after a maximal inspiration, while sitting, compared to the FVC for healthy adults of comparable age, sex, race and height in the National Health and Nutrition Examination Survey (NHANES) III.
(iv) Subject’s Global Impression of Change (SGIC) overall physical well-being score [18–20]: the patient answers this question using a 7-point rating scale that ranges from ‘1 = very much worse’ to ‘7 = very much improved’. In this study, answers were grouped into 3 categories: improving (patient scored ‘somewhat improved’, ‘much improved’, or ‘very much improved’); stable (patient scored ‘no change’); or declining (patient scored ‘somewhat worse’, ‘much worse’, or ‘very much worse’).
(v) PROMIS® Physical Function short form 20a [21, 22]: (PROMIS PF): This score is obtained after the patient completes a questionnaire with 20 questions on physical function, which the patient can score from unable to do (1) to be able to do without any difficulties or limitations (5). Hence, the score ranges between 20 and 100, with a higher score indicating better physical functioning.

### Statistical analyses

Three anchors were considered for the anchor-based approach: the SGIC, the PROMIS PF, and the FVC (% predicted), since we hypothesized that those potential anchors correlate well with 6MWD, and since established thresholds to define improvement exist for them. To check whether the basic prerequisite for an anchor was satisfied, Pearson’s correlation coefficient was calculated for the 6MWD (% predicted) change from baseline at Week 52 and SGIC overall physical well-being, and for the changes from baseline at Week 52 of 6MWD (% predicted) and PROMIS PF, and of 6MWD (% predicted) and FVC (% predicted).

To determine the MCID using SGIC as an anchor, the mean change (and SD) in 6MWD (% predicted) was calculated for each SGIC overall physical well-being score at Week 52. The MCID was defined as the mean change in 6MWD (% predicted) in patients who had reported that their SGIC overall physical well-being was stable to very much improved (score 4 to 7). In sensitivity analyses, the MCID was defined as the mean change in 6MWD (% predicted) in patients who had somewhat improved to very much improved according to their SGIC overall well-being score at Week 52 (score 5 to 7; MCID≥5), as the mean change in 6MWD % predicted in patients who were stable according to their SGIC overall well-being score at Week 52 (score 4; MCID=4), and as the mean change in 6MWD % predicted in patients who had somewhat improved according to their SGIC overall well-being score at Week 52 (score 5; MCID=5).

The MCID using PROMIS PF as an anchor was defined as the mean change (and SD) in 6MWD (% predicted) for patients with a non-negative PROMIS PF change from baseline at Week 52. A change of 2 to 6 points for PROMIS measures is considered to be important according to a systematic review of MCID estimates of PROMIS measures [23]; therefore, PROMIS PF change from baseline at Week 52 of at least 2 points (MCID≥2) and of at least 4 points (MCID≥4) was also considered in sensitivity analyses to define the MCID.

The MCID using FVC (% predicted) as an anchor was defined as the mean change (and SD) in 6MWD (% predicted) for patients with an FVC (% predicted) change from baseline at Week 52 larger or equal to 3%, which is in the range of MCIDs derived for idiopathic pulmonary fibrosis [24], and in line with clinically meaningful thresholds derived for LOPD [25].

The distribution-based MCID was defined as 1/3, 0.4 or 1/2 of the SD of 6MWD % predicted at baseline and at change from baseline at Week 52 [4, 26–28]. The anchor- and distribution-based MCIDs were calculated for the overall patient population of PROPEL and for the following subgroups, in order to explore possible covariates influencing the MCID: patients with baseline 6MWD < 150 m, < 300 m, < 400 m, < 450 m, and ≥ 450 m, based on two considerations: < 150 m, < 300 m, < 450 m and ≥ 450 m are multiples of the threshold of < 150 m that defines patients with a decreased survival prognosis [29], and since there were many patients who were able to walk between 300 and 400 m at baseline, the subgroup of < 400 m was added. To explore the impact of the chosen subgroups in a sensitivity analysis, MCIDs for subgroups of patients with baseline 6MWD < 150 m, ≥ 150 m and < 300 m, ≥ 300 m and < 450 m, and ≥ 450 m were calculated, respectively. MCIDs were also calculated in body mass index (BMI) subgroups: underweight (baseline BMI < 18.5 kg/m^2^), normal weight (baseline BMI ≥ 18.5 kg/m^2^ and < 25 kg/m^2^), overweight (baseline BMI ≥ 25 kg/m^2^ and < 30 kg/m^2^), and obese (baseline BMI ≥ 30 kg/m^2^) [30]. Additionally, we explored MCIDs in subgroups of patients with medical history or comorbidities relevant to walking ability and LOPD in general: patients having had a knee or hip surgery in the past, patients with heart failure, and patients with chronic obstructive pulmonary disease (COPD).

Like the MCID calculations for 6MWD (% predicted), we calculated anchor-based and distribution-based MCIDs for 6MWD (m), for the overall PROPEL population, and for subgroups of baseline 6MWD (m).

## Results

### Study population

Patients were randomly assigned 2:1 to cipaglucosidase alfa plus miglustat or alglucosidase alfa plus placebo treatment groups in the PROPEL study. The baseline characteristics of the 123 patients with LOPD in the intent-to-treat population of the PROPEL study have been described previously [16]. Briefly, 55% of the patients were female, the overall mean age was 46.8 years (SD 13.3), 85% of the patients were White, 77% had been treated previously with ERT, and most of the patients had the intervening sequence splice site mutation c.-32-13 T > G. For the cipaglucosidase alfa plus miglustat and the alglucosidase alfa plus placebo treatment groups, the mean/median baseline 6MWD (m) was 357.9/359.5 m (SD 111.8) and 351.0/365.5 m (SD 121.3), the mean/median baseline 6MWD (% predicted) was 57.8/59.2% (SD 15.8), and 56.0/56.1% (SD 17.3), and the mean/median baseline sitting FVC (% predicted) was 70.7/70.0% (SD 19.6) and 69.7/71.0% (SD 21.5), respectively.

### Anchor-based MCID

6MWD (% predicted) change from baseline at Week 52 was significantly positively correlated with PROMIS PF change from baseline at Week 52 (*R* = 0.374, *p* < 0.0001) and with SGIC at Week 52 (*R* = 0.304, *p* = 0.00086), and it was positively correlated with FVC (% predicted) change from baseline at Week 52 (*R* = 0.175, *p* = 0.05527, Supplementary Fig. S1).

**Fig. 1:**
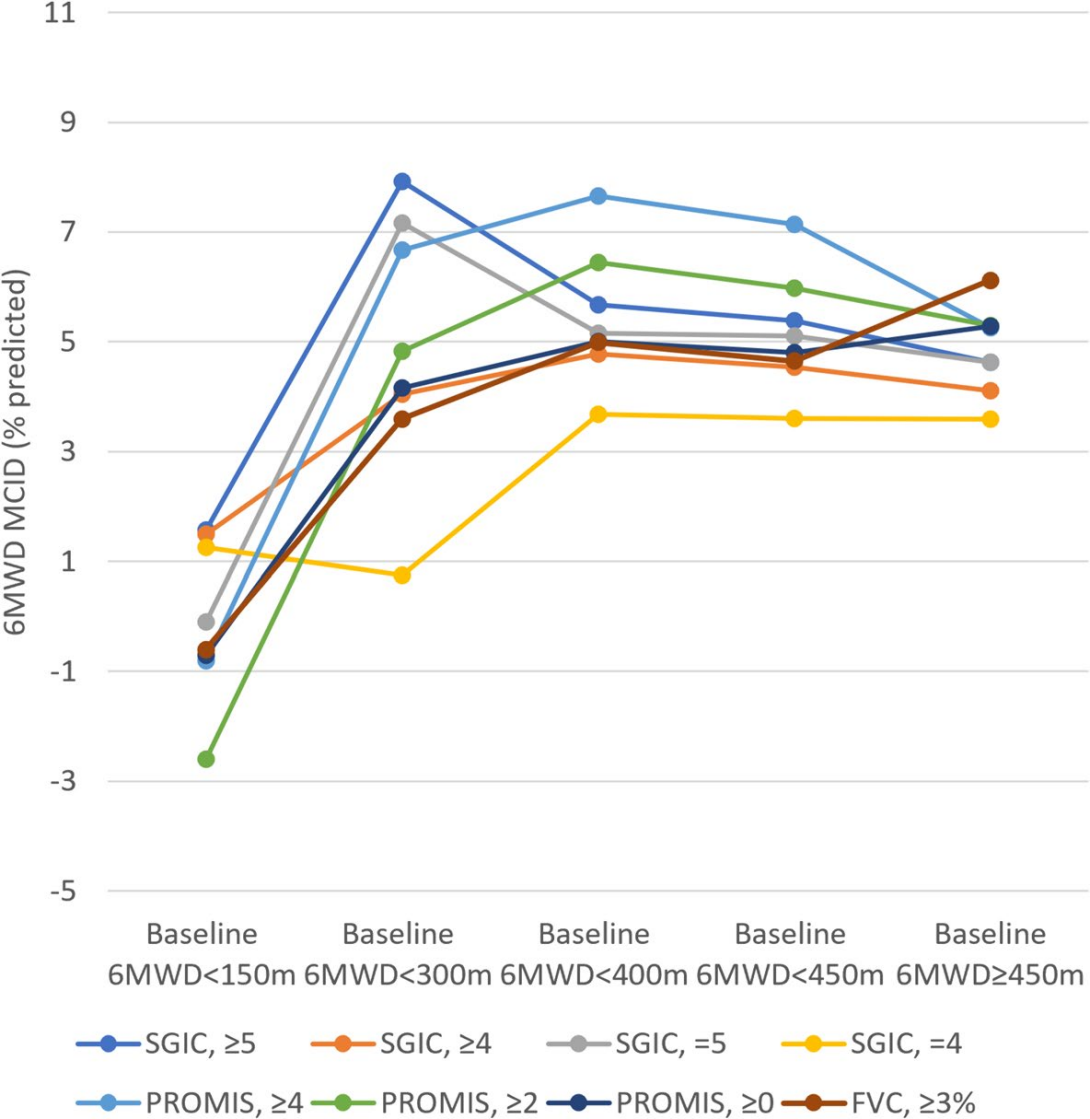
Anchor-based MCIDs for 6MWD (% predicted) for subgroups of patients with baseline 6MWD < 150 m, < 300 m, < 400 m, < 450 m, and ≥ 450 m. Abbreviations: *6MWD* 6-min walk distance, *m* meter, *FVC* forced vital capacity, *FVC* ≥ *3%* MCID based on patients with an FVC (% predicted) change from baseline of ≥ 3% at Week 52, *MCID* minimal clinically important difference, *PROMIS* Patient-Reported Outcome Measures Information System, *PROMIS* ≥ *0* MCID based on patients with a PROMIS Physical Function short form 20a (PF) change from baseline of ≥ 0 points at Week 52, *PROMIS* ≥ *2* MCID based on patients with a PROMIS PF change from baseline of ≥ 2 points at Week 52, *PROMIS* ≥ *4* MCID based on patients with a PROMIS PF change from baseline of ≥ 4 points at Week 52, *SGIC* Subject’s Global Impression of Change, *SGIC* ≥ *4* MCID based on patients with an SGIC of ≥ 4 points at Week 52, *SGIC* ≥ *5* MCID based on patients with an SGIC of ≥ 5 points at Week 52, *SGIC* = *4* MCID based on patients with an SGIC of = 4 points at Week 52, *SGIC* = *5* MCID based on patients with an SGIC of = 5 points

In the overall PROPEL population, the 6MWD (% predicted) MCID was 4.93%, 4.45%, and 4.85%, with PROMIS PF, SGIC, and FVC as anchors, respectively. The anchor-based 6MWD (% predicted) MCIDs in subgroups of baseline severity are summarized in Table 1. 6MWD MCIDs increased with more meters walked at baseline until a plateau was reached. Varying the definitions of the anchor-based 6MWD (% predicted) MCIDs in the sensitivity analysis (i.e., using ≥ 2- or ≥ 4- -point change from baseline in PROMIS PF to define the MCID or using an SGIC overall well-being score of ≥ 5, = 4 or = 5 to define the MCID) revealed that those MCIDs were consistently higher than the 6MWD (% predicted) MCIDs shown in Table 1, except when the MCID was defined by an SGIC overall well-being score = 4 (Supplementary Table S1). Varying the subgroups in the sensitivity analysis yielded similar results, i.e., 6MWD (% predicted) MCIDs increased with baseline meters walked, reaching a plateau (Supplementary Table S2). The increase in anchor-based 6MWD MCIDs with more meters walked at baseline is also illustrated in Fig. 1 (Supplementary Fig. S2 for the sensitivity analysis).

**Table 1:** Anchor-based MCID for 6MWD (% predicted)

**Subgroup**		**PROMIS PF as Anchor MCID**_**≥0**_	**SGIC as Anchor MCID**_**≥4**_	**FVC as Anchor MCID**_**≥3%**_
Overall	N	71	94	22
	MCID (SD), %	4.93 (7.05)	4.45 (6.84)	4.85 (6.99)
**Baseline 6MWD subgroups**				
Baseline 6MWD < 150 m	N	4	4	3
	MCID (SD), %	-0.74 (4.89)	1.49 (2.40)	-0.63 (2.07)
Baseline 6MWD < 300 m	N	20	24	9
	MCID (SD), %	4.16 (6.79)	4.04 (6.34)	3.59 (6.16)
Baseline 6MWD < 400 m	N	46	65	18
	MCID (SD), %	5.00 (8.28)	4.78 (7.69)	4.99 (7.33)
Baseline 6MWD < 450 m	N	52	74	19
	MCID (SD), %	4.80 (7.89)	4.54 (7.33)	4.65 (7.27)
Baseline 6MWD ≥ 450 m	N	19	20	3
	MCID (SD), %	5.28 (4.10)	4.11 (4.77)	6.12 (5.82)
**BMI subgroups**				
Underweight	N	5	6	1
	MCID (SD), %	4.00 (7.56)	3.33 (6.95)	0.50 (NA)
Normal weight	N	36	45	11
	MCID (SD), %	7.20 (7.02)	5.87 (7.14)	5.95 (5.70)
Overweight	N	17	25	6
	MCID (SD), %	1.94 (5.95)	3.01 (6.66)	6.34 (10.23)
Obese	N	13	18	4
	MCID (SD), %	2.92 (6.87)	3.24 (6.09)	0.66 (4.51)
**Comorbidities subgroups**				
Having had knee or hip surgery in the past	N	8	8	1
	MCID (SD), %	9.13 (6.40)	9.13 (6.40)	7.25 (NA)
COPD	N	3	2	1
	MCID (SD), %	0.37 (3.70)	-0.83 (4.35)	2.25 (NA)
Heart failure	N	1	1	0
	MCID (SD), %	-3.90 (NA)	-3.90 (NA)	-

Underweight: baseline BMI < 18.5 kg/m^2^; normal weight: baseline BMI ≥ 18.5 kg/m^2^ and < 25 kg/m^2^; overweight: baseline BMI ≥ 25 kg/m^2^ and < 30 kg/m^2^; and obese: baseline BMI ≥ 30 kg/m^2^

Abbreviations: *6MWD* 6-min walk distance, *BMI* body mass index, *COPD* chronic obstructive pulmonary disease, *FVC* forced vital capacity, *m* meter, *MCID* minimal clinically important difference, *NA* not applicable, *PROMIS PF* PROMIS^®^ Physical Function short form 20a, *SD* standard deviation, *SGIC* Subject’s Global Impression of Change

The results were similar when anchor-based MCIDs were calculated for 6MWD (meters) using varying MCID thresholds and subgroup definitions (Supplementary Table S3). The anchor-based MCIDs varied between 23.70 m to 40.11 m for the overall PROPEL population, depending on the method, were lower for baseline 6MWD < 150 m (-14.33 to 11.53 m) and increased with baseline meters walked, reaching a plateau.

Subgroup analysis based on baseline BMI levels usually showed highest 6MWD (% predicted) MCIDs in the normal weight subgroup, ranging from 5.87% to 7.20%, and lowest 6MWD (% predicted) MCIDs in the obese subgroup with MCIDs ranging from 0.66% to 3.24% (Table 1). Sensitivity analyses varying the MCID thresholds for the BMI subgroups revealed similar results to those shown in Table 1 i.e., the highest MCIDs were generally observed in the normal BMI subgroup and lowest MCIDs in the obese subgroup (Supplementary Table S1).

Analyses of medical history or comorbidities subgroups revealed higher anchor-based MCIDs in the subgroup of patients having had a knee or hip surgery in the past than in the overall PROPEL population, ranging from 6.74% to 14.10% (Table 1 and Supplementary Table S1). Subgroups of COPD and heart failure had too small sample sizes to draw robust conclusions.

### Distribution-based MCID

**Fig. 2:**
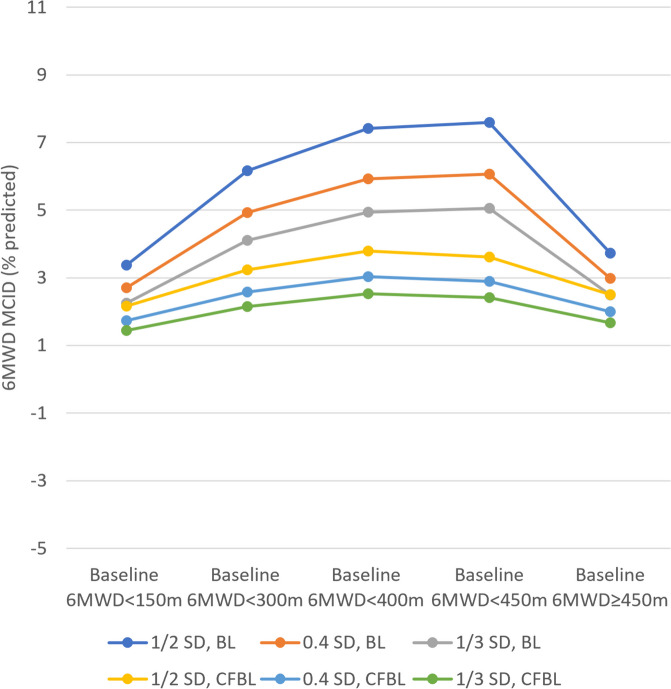
Distribution-based MCID for 6MWD (% predicted) for subgroups of patients with baseline 6MWD < 150 m, < 300 m, < 400 m, < 450 m, and ≥ 450 m. Abbreviations: *6MWD* 6-min walk distance, *m* meter, *MCID* minimal clinically important difference, *SD* standard deviation, *SD BL* MCID based on the standard deviation at baseline, *SD CFBL* MCID based on the standard deviation at change from baseline at Week 52

Distribution-based MCIDs for 6MWD (% predicted) in the overall PROPEL population were 8.11% (1/2 SD), 6.48% (0.4 SD), and 5.40% (1/3 SD) when baseline 6MWD (% predicted) values were considered, and 3.4% (1/2 SD), 2.72% (0.4 SD), and 2.27% (1/3 SD) when the 6MWD (% predicted) change from baseline at Week 52 values were considered for the MCID calculation. The MCIDs for 6MWD (% predicted) using a distribution-based approach​ are summarized in Table 2. The lowest MCIDs (% predicted) were observed in patients with impaired walking abilities (i.e., patients having baseline 6MWD < 150 m), with MCIDs ranging from 1.44 (1/3 SD of the change from baseline at Week 52 values) to 3.37 (1/2 SD of the baseline values). The MCIDs increased with more meters walked at baseline until reaching a plateau and even a decline for the subgroup of patients with baseline 6MWD ≥ 450 m. Similar results were observed in sensitivity analyses applying different subgroup definitions (Supplementary Table S4). The increase in distribution-based 6MWD MCIDs with more meters walked at baseline is also illustrated in Fig. 2 (Supplementary Fig. S3 for the sensitivity analysis).

**Table 2:** Distribution-based MCID for 6MWD (% predicted)

		**Baseline**			**Change from baseline**		
**Subgroup**	**N**	**1/2 SD MCID**	**0.4 SD MCID**	**1/3 SD MCID**	**1/2 SD MCID**	**0.4 SD MCID**	**1/3 SD MCID**
Overall	122	8.11	6.48	5.40	3.40	2.72	2.27
**Baseline 6MWD subgroups**							
Baseline 6MWD < 150 m	8	3.37	2.70	2.25	2.16	1.73	1.44
Baseline 6MWD < 300 m	33	6.16	4.93	4.11	3.23	2.58	2.15
Baseline 6MWD < 400 m	84	7.41	5.93	4.94	3.79	3.03	2.53
Baseline 6MWD < 450 m	95	7.59	6.07	5.06	3.61	2.89	2.41
Baseline 6MWD ≥ 450 m	27	3.73	2.98	2.49	2.50	2.00	1.67
**BMI subgroups**							
Underweight	7	6.49	5.19	4.33	3.47	2.78	2.32
Normal weight	63	7.99	6.39	5.33	3.63	2.91	2.42
Normal weight	30	7.75	6.20	5.17	3.24	2.59	2.16
Obese	22	9.57	7.65	6.38	2.91	2.33	1.94
**Comorbidities subgroups**							
Having had knee or hip surgery in the past	8	7.32	5.85	4.88	3.2	2.56	2.13
COPD	3	8.63	6.9	5.75	1.85	1.48	1.23
Heart failure	1	NA	NA	NA	NA	NA	NA

Underweight: baseline BMI < 18.5 kg/m^2^; normal weight: baseline BMI ≥ 18.5 kg/m^2^ and < 25 kg/m^2^; overweight: baseline BMI ≥ 25 kg/m^2^ and < 30 kg/m^2^; and obese: baseline BMI ≥ 30 kg/m^2^

Abbreviations: *6MWD* 6-min walk distance, *BMI* body mass index, *COPD* chronic obstructive pulmonary disease, *m* meter, *MCID*, minimal clinically important difference, *NA* not applicable, *SD* standard deviation

Similar results were observed for the distribution-based MCID for 6MWD when calculated in meters, with a range of 38.1 to 57.2 m for the overall PROPEL population. The MCIDs were lower for baseline 6MWD < 150 m (7.5 to 11.3 m) and increased with baseline meters walked, reaching a plateau (Supplementary Table S5).

Distribution-based MCIDs in baseline BMI subgroups were highest in the subgroup with a normal weight, except for the MCIDs when using baseline 6MWD (% predicted) values to derive the MCID, where the highest values were observed in the obese subgroup. Inspection of the distribution of the 6MWD (% predicted) values in this subgroup revealed that unexpectedly, many patients had low and high baseline 6MWD (% predicted) values, leading to a larger SD and thus a larger MCID in that subgroup.

Analyses of medical history or comorbidities subgroups revealed similar distribution-based MCIDs in the subgroup of patients having had a knee or hip surgery in the past as compared to MCIDs for the overall PROPEL population, ranging from 2.13% to 7.32% (Table 2). Subgroups of COPD and heart failure had too small sample sizes to draw robust conclusions.

## Discussion

The MCIDs for 6MWD (% predicted and meters) for patients with LOPD from the PROPEL study were derived using anchor- and distribution-based approaches. We have sought to address the evidence gap of MCID for 6MWD (% predicted and meters) in LOPD by applying different analysis methods using data from the PROPEL study.

The calculated MCIDs varied widely and were dependent upon the choice of analysis method and the subgroup, as defined by baseline walking distance, BMI, or medical history/comorbidities. For the overall PROPEL population, a range of 2.27% to 8.11% was determined, depending on the method used. For patients with impaired walking ability, with a baseline 6MWD of less than 150 m, a range of -0.74% to 3.37% was calculated as MCID for 6MWD (% predicted), indicating that stability or even a small decline in 6MWD could be considered as improvement for patients in a severe and potentially rapidly progressing phase of the disease. On the other hand, MCIDs increased with more meters walked at baseline, reaching a plateau, which suggests that patients in a better disease stage would require a larger increase of 6MWD to be considered an improvement. This trend was also observed for several sensitivity analyses when varying the method of calculating the MCID and varying the definition of the subgroups of baseline severity.

Inspection of MCIDs in baseline BMI subgroups generally indicated the highest MCID 6MWD (% predicted) for patients with normal weight, and the lowest MCID for obese patients. MCIDs for patients having had a knee or hip surgery in the past were similar or higher than MCIDs for the overall PROPEL population, suggesting that surgical interventions of this type can lead to improved mobility post-surgery for LOPD patients, and these patients experience a larger increase in 6MWD as an improvement with an associated larger MCID for 6MWD compared to the general PROPEL population.

Applying the same approaches to 6MWD in meters revealed similar patterns; the MCID was 23.7 to 57.2 m in the overall PROPEL population, depending on the method used. This is in line with MCID values reported in similar diseases: a meta-analysis of 11 studies in neuromuscular diseases revealed an MCID for 6MWD of 22.2–55.5 m [11]. The MCID in meters was lowest in the subgroup of patients with baseline 6MWD < 150 m (-2.1 to 11.3 m) and increased with more baseline meters walked until a plateau was reached.

Some researchers recommend not to calculate MCIDs in subgroups defined by stratification of the baseline score, since this may lead to biased MCID results for those subgroups [8]. However, we found similar results for different stratifications of the baseline 6MWD (m) in the sensitivity analyses, and exploration of other subgroups that are reflective of baseline severity revealed a similar pattern: patients with a BMI outside the normal weight range generally had lower MCIDs, and patients with a knee or hip surgery in the past suggesting better walking ability than the overall PROPEL population showed higher MCIDs. Our analyses highlight that MCID depends on disease status. For a progressive disease such as LOPD, the findings suggest that a single MCID that can be applied to all patients can be misleading. Instead, when estimating MCIDs, we suggest that a range of possible MCIDs should be considered that incorporates the degree of the patient’s disease progression at baseline.

The study has some limitations. First, the patient-level data used to derive 6MWD MCIDs were from a single trial, the PROPEL study, in LOPD, thus results may not be generalizable to the overall LOPD patient population. However, the PROPEL study is a relatively large randomized clinical trial for this disease area and reflective of the general LOPD population in terms of pre-treatment with ERTs, since it contains a mix of ERT-naive and -experienced patients (23% vs. 77%, respectively) that is comparable to the distribution observed in ongoing Pompe disease registry studies in which ~ 78–80% of patients have received ERT previously [31, 32].

Second, the patient numbers in the subgroups of fewest meters walked at baseline (6MWD < 150 m), were small, thus, the resulting 6MWD MCIDs were driven by a few patients, and might not be robust and transferable to another patient population.

Third, correlations of 6MWD (% predicted) and FVC (% predicted) changes from baseline at Week 52 were positive but not significant, therefore, MCID results using FVC as an anchor might not be as robust as MCID results using PROMIS PF or SGIC as anchors.

## Conclusions

This study provides a range of 6MWD MCIDs for LOPD, with lower MCIDs for more severe patients. While this study highlights the challenges associated with calculating MCID in a progressive disease such as LOPD, and how different approaches (anchor- versus distribution-based) can lead to varying estimates, it also contributes to the scientific and evidence-based knowledge of MCIDs for 6MWD in this disease. Moreover, the findings support the contention that a single MCID applied to a population with different stages of disease may be misleading and that a stratified MCID approach is more informative: patients with severe baseline conditions should not be expected to benefit to the same absolute extent from a therapy as patients with mild or early stage, but that benefit can and should still be considered meaningful. This may be highly relevant for other neuromuscular diseases as well.

### Supplementary Information

**Additional file 1:** **Supplementary Figure S1.** Correlation of 6MWD (% predicted) with PROMIS PF, SGIC and FVC (% predicted). **Supplementary Table S1.** Anchor-based MCID for 6MWD (% predicted), sensitivity analysis, MCID definition. **Supplementary Table S2.** Anchor-based MCID for 6MWD (% predicted), sensitivity analysis, subgroup definition. **Supplementary Figure S2.** Anchor-based MCIDs for 6MWD (% predicted) for subgroups of patients with baseline 6MWD <150 meters, ≥150 meters and <300 meters, ≥300 meters and <450 meters, and ≥ 450 meters. **Supplementary Table S3.** Anchor-based MCID for 6MWD (m). **Supplementary Table S4.** Distribution-based MCID for 6MWD (% predicted), sensitivity analysis, subgroup definition. **Supplementary Figure S3.** Distribution-based MCIDs for 6MWD (% predicted) for subgroups of patients with baseline 6MWD <150 meters, ≥150 meters and <300 meters, ≥300 meters and <450 meters, and ≥ 450 meters. **Supplementary Table S5.** Distribution-based MCID for 6MWD (meters).

## Data Availability

Data sharing proposals and requests for data of the PROPEL study will be reviewed on a case-by-case basis. Requests for data should be addressed to Mitchell Goldman at mgoldman@amicusrx.com. Requests will be reviewed by a medical steering committee.

## Abbreviations

BMI: Body mass index
COPD: Chronic obstructive pulmonary disease
ERT: Enzyme replacement therapy
FVC: Forced vital capacity
HTA: Health Technology Assessment
LOPD: Late-onset Pompe disease
MCID: Minimal clinically important difference
NA: Not applicable
NHANES: National Health and Nutrition Examination Survey
PROMIS: Patient-Reported Outcomes Measurement Information System
PROMIS PF: PROMIS^®^ Physical Function short form 20a
SD: Standard deviation
SGIC: Subject’s Global Impression of Change
6MWD: 6-min walk distance
