# Supplementary material for: Minimal clinically important differences in six-minute walking distance in late-onset Pompe disease
Source: Orphanet J Rare Dis. 2024 Apr 11;19:154. doi: 10.1186/s13023-024-03156-3 (PMC11008008; doi:10.1186/s13023-024-03156-3)
Supplement: Supplementary file 1 — Additional file 1: Supplementary Figure S1. Correlation of 6MWD (% predicted) with PROMIS PF, SGIC and FVC (% predicted). Supplementary Table S1. Anchor-based MCID for 6MWD (% predicted), sensitivity analysis, MCID definition. Supplementary Table S2. Anchor-based MCID for 6MWD (% predicted), sensitivity analysis, subgroup definition. Supplementary Figure S2. Anchor-based MCIDs for 6MWD (% predicted) for subgroups of patients with baseline 6MWD <150 meters, ≥150 meters and <300 meters, ≥300 meters and <450 meters, and ≥ 450 meters. Supplementary Table S3. Anchor-based MCID for 6MWD (m). Supplementary Table S4. Distribution-based MCID for 6MWD (% predicted), sensitivity analysis, subgroup definition. Supplementary Figure S3. Distribution-based MCIDs for 6MWD (% predicted) for subgroups of patients with baseline 6MWD <150 meters, ≥150 meters and <300 meters, ≥300 meters and <450 meters, and ≥ 450 meters. Supplementary Table S5. Distribution-based MCID for 6MWD (meters). [file 13023_2024_3156_MOESM1_ESM.pdf]

Supplementary material

Supplementary Fig. S1 Correlation of 6MWD (% predicted) with PROMIS PF, SGIC and FVC (% predicted)

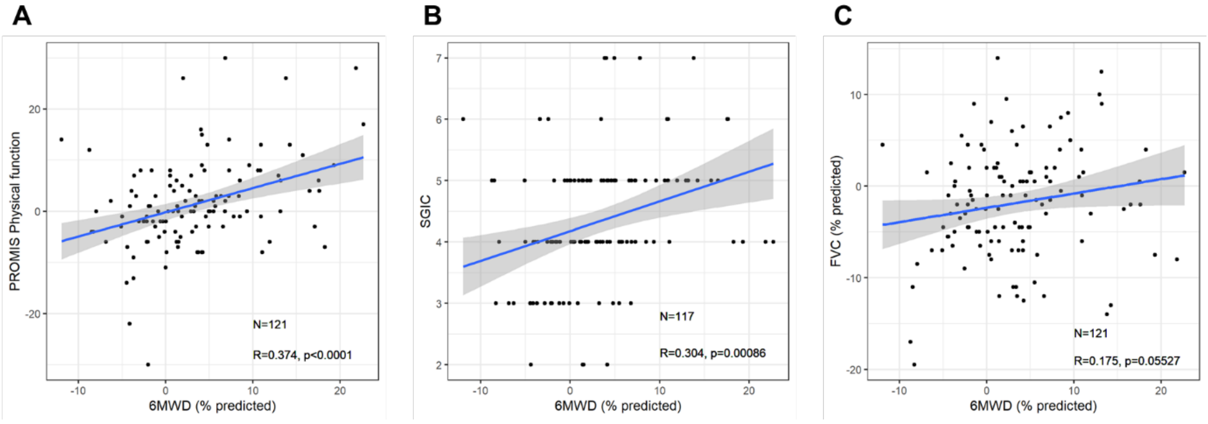

(**A**) Correlation of 6MWD (% predicted) change from baseline at Week 52 with PROMIS PF change from baseline at Week 52, (**B**) Correlation of 6MWD (% predicted) change from baseline at Week 52 with SGIC at Week 52, (**C**) Correlation of 6MWD (% predicted) change from baseline at Week 52 with FVC (% predicted) change from baseline at Week 52.

Abbreviations: 6MWD, 6-minute walk distance; FVC, forced vital capacity; PROMIS PF, PROMIS® Physical Function short form 20a; SGIC, Subject's Global Impression of Change.

Supplementary Table S1 Anchor-based MCID for 6MWD (% predicted), sensitivity analysis, MCID definition

Subgroup		PROMIS PF as Anchor		SGIC as Anchor		
		PROMIS $MCID_{\geq 4}$	PROMIS $MCID_{\geq 2}$	SGIC $MCID_{=5}$	SGIC $MCID_{\geq 5}$	SGIC $MCID_{=4}$
Overall	N	39	52	36	49	45
	MCID (SD), %	6.66 (8.20)	5.82 (7.54)	4.97 (6.34)	5.23 (6.92)	3.60 (6.73)
		**Baseline 6MWD subgroups**				
Baseline 6MWD<150m	N	2	3	2	3	1
	MCID (SD), %	-0.80 (2.90)	-2.63 (3.78)	-0.13 (0.25)	1.57 (2.94)	1.25 (NA)
Baseline 6MWD<300m	N	8	14	9	11	13
	MCID (SD), %	6.67 (8.15)	4.83 (7.17)	7.17 (5.90)	7.92 (6.22)	0.75 (4.39)
Baseline 6MWD<400m	N	24	34	24	36	29
	MCID (SD), %	7.66 (9.82)	6.45 (8.72)	5.15 (6.72)	5.67 (7.26)	3.68 (8.19)
Baseline 6MWD<450m	N	29	40	26	39	35
	MCID (SD), %	7.14 (9.01)	5.98 (8.22)	5.10 (6.45)	5.38 (7.12)	3.60 (7.54)
Baseline 6MWD≥450m	N	10	12	10	10	10
	MCID (SD), %	5.26 (5.33)	5.30 (4.86)	4.63 (6.38)	4.63 (6.38)	3.59 (2.60)
		**BMI subgroups**				
Underweight	N	4	5	4	4	2
	MCID (SD), %	3.29 (8.53)	4.00 (7.56)	5.78 (7.34)	5.78 (7.34)	-1.55 (2.90)
Normal weight	N	20	26	17	24	21

	MCID (SD), %	10.20 (7.26)	8.37 (7.45)	5.42 (7.00)	6.04 (7.06)	5.68 (7.40)
Overweight	N	7	10	8	11	14
	MCID (SD), %	2.41 (7.51)	2.96 (6.61)	5.73 (3.92)	5.06 (6.85)	1.40 (6.28)
Obese	N	8	11	7	10	8
	MCID (SD), %	3.23 (8.31)	3.22 (7.42)	2.56 (7.10)	3.24 (7.16)	3.25 (4.90)
	**Comorbidities subgroups**					
Having had knee or hip surgery in the past	N	5	7	4	6	2
	MCID (SD), %	11.59 (6.82)	10.22 (6.04)	6.74 (3.93)	7.47 (4.48)	14.10 (10.96)
COPD	N	2	3	1	1	1
	MCID (SD), %	-0.58 (4.70)	0.37 (3.70)	2.25 (NA)	2.25 (NA)	-3.90 (NA)
Heart failure	N	1	1	0	0	1
	MCID (SD), %	-3.90 (NA)	-3.90 (NA)	-	-	-3.90 (NA)

Abbreviations: 6MWD, 6-minute walk distance; BMI, body mass index; m, meter; MCID, minimal clinically important difference; PROMIS PF, PROMIS® Physical Function short form 20a; SD, standard deviation; SGIC, Subject’s Global Impression of Change.

Underweight: baseline BMI <18.5 kg/m^2; normal weight: baseline BMI ≥18.5 kg/m2 and <25 kg/m^2; overweight: baseline BMI ≥25 kg/m2 and <30 kg/m^2; and obese: baseline BMI ≥30 kg/m^2.

Supplementary Table S2 Anchor-based MCID for 6MWD (% predicted), sensitivity analysis, subgroup definition

**Subgroup**		**PROMIS PF as Anchor MCID$_{\geq 0}$**	**SGIC as Anchor MCID$_{\geq 4}$**	**FVC as Anchor MCID$_{\geq 3\%}$**
Overall	N	71	94	22
	MCID (SD), %	4.93 (7.05)	4.45 (6.84)	4.85 (6.99)
Baseline 6MWD<150m	N	4	4	3
	MCID (SD), %	-0.74 (4.89)	1.49 (2.40)	-0.63 (2.07)
150m≤Baseline 6MWD<300m	N	16	20	6
	MCID (SD), %	5.38 (6.76)	4.55 (6.79)	5.70 (6.55)
300m≤Baseline 6MWD<450m	N	32	50	10
	MCID (SD), %	5.21 (8.58)	4.78 (7.80)	5.61 (8.36)
Baseline 6MWD≥450m	N	19	20	3
	MCID (SD), %	5.28 (4.10)	4.11 (4.77)	6.12 (5.82)

Abbreviations: 6MWD, 6-minute walk distance; FVC, forced vital capacity; m, meter; MCID, minimal clinically important difference; PROMIS PF, PROMIS® Physical Function short form 20a; SD, standard deviation; SGIC, Subject's Global Impression of Change.

Supplementary Fig. S2 Anchor-based MCIDs for 6MWD (% predicted) for subgroups of patients with baseline 6MWD <150 meters, ≥150 meters and <300 meters, ≥300 meters and <450 meters, and ≥ 450 meters.

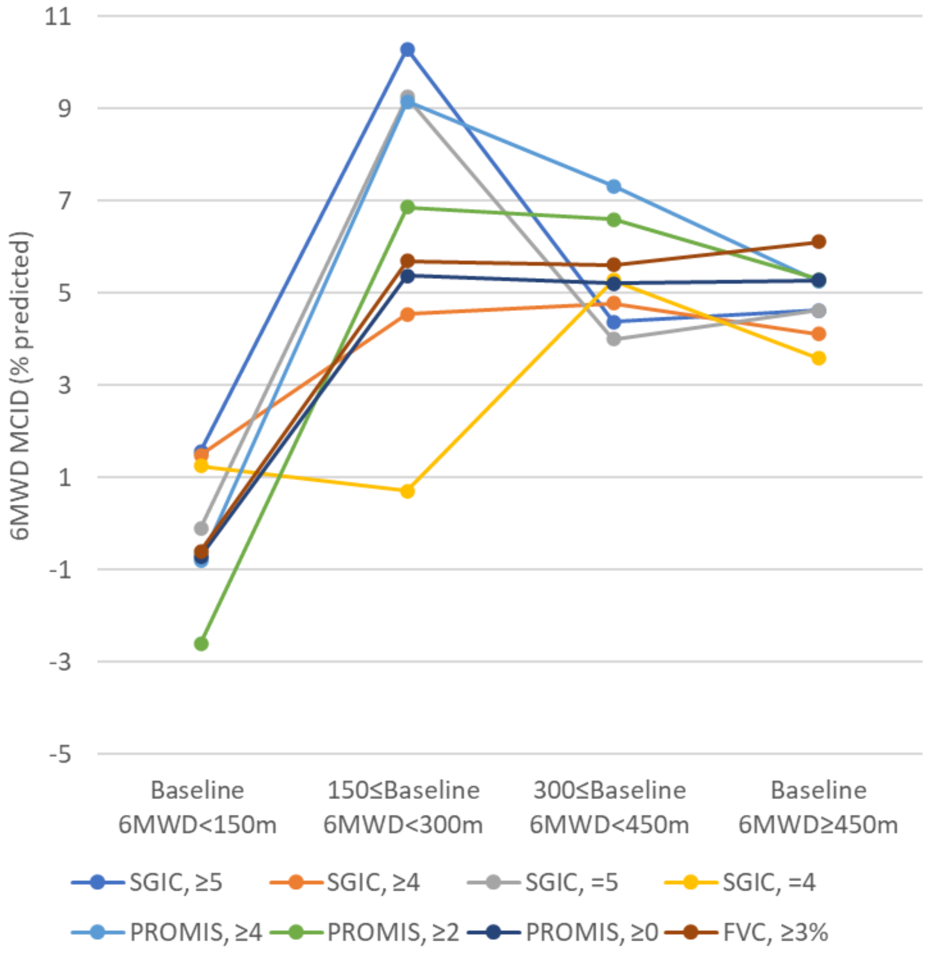

Abbreviations: 6MWD, 6-minute walk distance; m, meter; FVC, forced vital capacity; FVC, ≥3%, MCID based on patients with an FVC (% predicted) change from baseline of ≥3% at Week 52; MCID, minimal clinically important difference; PROMIS, patient-reported outcome measures information system; PROMIS, ≥0, MCID based on patients with a PROMIS PF change from baseline of ≥0 points at Week 52; PROMIS, ≥2, MCID based on patients with a PROMIS PF change from baseline of ≥2 points at Week 52; PROMIS, ≥4, MCID based on patients with a PROMIS PF change from baseline of ≥4 points at Week 52; SGIC, Subject's Global Impression of

Change; SGIC, ≥4, MCID based on patients with an SGIC of ≥4 points at Week 52; SGIC, ≥5, MCID based on patients with an SGIC of ≥5 points at Week 52; SGIC, =4, MCID based on patients with an SGIC of =4 points at Week 52; SGIC, =5, MCID based on patients with an SGIC of =5 points

Supplementary Table S3 Anchor-based MCID for 6MWD (m)

**Subgroup**		**PROMIS PF as Anchor**			**SGIC as Anchor**	
		**PROMIS $MCID_{\geq 0}$**	**PROMIS $MCID_{\geq 2}$**	**PROMIS $MCID_{\geq 4}$**	**SGIC $MCID_{\geq 4}$**	**SGIC $MCID_{\geq 5}$**
Overall	N	71	52	39	94	49
	MCID (SD), m	28.2 (44.5)	33.85 (47.43)	40.11 (51.44)	23.7 (42.6)	29.11 (43.66)
Baseline 6MWD<150m	N	4	3	2	4	8
	MCID (SD), m	-2.1 (30.9)	-14.33 (23)	-2.75 (15.91)	10.8 (16.5)	11.53 (20.08)
Baseline 6MWD<300m	N	20	14	8	24	11
	MCID (SD), m	23.3 (40.8)	26.50 (42.42)	39.86 (47.90)	22.1 (37.7)	46.80 (37.76)
Baseline 6MWD<400m	N	46	34	24	65	36
	MCID (SD), m	29.4 (51.8)	38.60 (54.18)	47.86 (60.64)	25.3 (47.4)	31.74 (45.29)
Baseline 6MWD<450m	N	52	40	29	74	39
	MCID (SD), m	28.1 (49.5)	35.56 (51.24)	44.34 (55.81)	24.2 (45.2)	30.29 (44.50)
150m≤baseline 6MWD<300m	N	16	11	6	20	8
	MCID (SD), m	29.58 (41.25)	37.64 (39.96)	54.07 (46.83)	24.33 (40.54)	60.03 (34.48)
300m≤baseline 6MWD<450m	N	32	26	21	50	28
	MCID (SD), m	31.15 (54.63)	40.43 (55.59)	46.05 (59.55)	25.23 (48.73)	23.81 (45.87)
Baseline 6MWD≥450m	N	19	12	10	20	10
	MCID (SD), m	28.33 (27.45)	28.16 (32.78)	27.84 (35.47)	21.62 (31.91)	24.48 (42.13)

Abbreviations: 6MWD, 6-minute walk distance; m, meter; MCID, minimal clinically important difference; PROMIS PF, PROMIS® Physical Function short form 20a; PROMIS $MCID_{\geq 0}$, MCID based on patients with a PROMIS PF change from baseline of ≥0 points at Week 52; PROMIS $MCID_{\geq 2}$, MCID based on patients with a PROMIS PF change from baseline of ≥2 points at Week 52; PROMIS $MCID_{\geq 4}$, MCID based on patients with a PROMIS PF change from baseline of ≥4 points at Week 52; SD, standard deviation; SGIC, Subject's Global Impression of Change; SGIC $MCID_{\geq 4}$, MCID based on patients with an SGIC of ≥4 points at Week 52; SGIC $MCID_{\geq 5}$, MCID based on patients with an SGIC of ≥5 points at Week 52.

Supplementary Table S4 Distribution-based MCID for 6MWD (% predicted), sensitivity analysis, subgroup definition

Subgroup	N	Baseline			Change from baseline at Week 52		
		**1/3 SD MCID**	**1/2 SD MCID**	**0.4 SD MCID**	**1/3 SD MCID**	**1/2 SD MCID**	**0.4 SD MCID**
Overall	122	5.4	8.11	6.48	2.27	3.4	2.72
Baseline 6MWD<150m	8	2.25	3.37	2.7	1.44	2.16	1.73
150m≤baseline 6MWD<300m	25	2.65	3.98	3.18	2.17	3.26	2.61
300m≤baseline 6MWD<450m	62	3.44	5.16	4.13	2.54	3.81	3.05
Baseline 6MWD≥450m	27	2.49	3.73	2.98	1.67	2.5	2

Abbreviations: 6MWD, 6-minute walk distance; m, meter; MCID, minimal clinically important difference; SD, standard deviation.

Supplementary Fig. S3 Distribution-based MCIDs for 6MWD (% predicted) for subgroups of patients with baseline 6MWD <150 meters, ≥150 meters and <300 meters, ≥300 meters and <450 meters, and ≥ 450 meters.

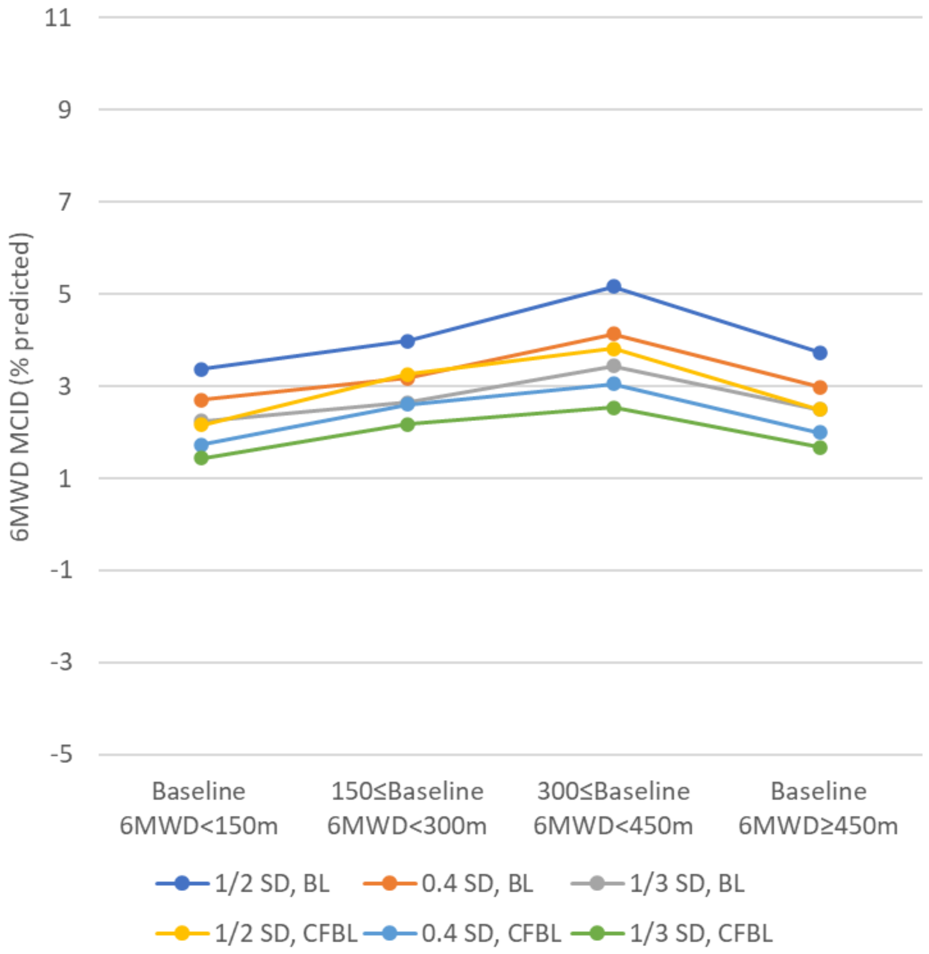

Abbreviations: 6MWD, 6-minute walk distance; m, meter; SD, standard deviation; SD, BL, MCID based on the standard deviation at baseline; SD, CFBL, MCID based on the standard deviation at change from baseline at Week 52.

Supplementary Table S5 Distribution-based MCID for 6MWD (meters)

Subgroup	N	1/3 SD MCID (m)	1/2 SD MCID (m)
Overall	122	38.1	57.2
Baseline 6MWD<150m	8	7.5	11.3
Baseline 6MWD<300m	33	22.2	33.2
Baseline 6MWD<400m	84	27.8	41.8
Baseline 6MWD<450m	95	29.1	43.7
150m≤baseline 6MWD<300m	25	15.1	22.6
300m≤baseline 6MWD<450m	62	12.1	18.2
Baseline 6MWD≥450m	27	13.7	20.6

Abbreviations: 6MWD, 6-minute walk distance; m, meter; MCID, minimal clinically important difference; SD, standard deviation.
